# Association Analysis Identifies *Melampsora* ×*columbiana* Poplar Leaf Rust Resistance SNPs

**DOI:** 10.1371/journal.pone.0078423

**Published:** 2013-11-13

**Authors:** Jonathan La Mantia, Jaroslav Klápště, Yousry A. El-Kassaby, Shofiul Azam, Robert D. Guy, Carl J. Douglas, Shawn D. Mansfield, Richard Hamelin

**Affiliations:** 1 Department of Forest and Conservation Sciences, University of British Columbia, Vancouver, British Columbia, Canada; 2 Department of Botany, University of British Columbia, Vancouver, British Columbia, Canada; 3 Department of Wood Science, University of British Columbia, Vancouver, British Columbia, Canada; 4 Natural Resources Canada, Laurentian Forestry Center, Québec, Canada; 5 Department of Dendrology and Forest Tree Breeding, Faculty of Forestry and Wood Sciences, Czech University of Life Sciences Prague, Kamýcká, Czech Republic; United States Department of Agriculture, Agricultural Research Service, United States of America

## Abstract

*Populus* species are currently being domesticated through intensive time- and resource-dependent programs for utilization in phytoremediation, wood and paper products, and conversion to biofuels. Poplar leaf rust disease can greatly reduce wood volume. Genetic resistance is effective in reducing economic losses but major resistance loci have been race-specific and can be readily defeated by the pathogen. Developing durable disease resistance requires the identification of non-race-specific loci. In the presented study, area under the disease progress curve was calculated from natural infection of *Melampsora* ×*columbiana* in three consecutive years. Association analysis was performed using 412 *P. trichocarpa* clones genotyped with 29,355 SNPs covering 3,543 genes. We found 40 SNPs within 26 unique genes significantly associated (permutated *P*<0.05) with poplar rust severity. Moreover, two SNPs were repeated in all three years suggesting non-race-specificity and three additional SNPs were differentially expressed in other poplar rust interactions. These five SNPs were found in genes that have orthologs in Arabidopsis with functionality in pathogen induced transcriptome reprogramming, Ca^2+^/calmodulin and salicylic acid signaling, and tolerance to reactive oxygen species. The additive effect of non-R gene functional variants may constitute high levels of durable poplar leaf rust resistance. Therefore, these findings are of significance for speeding the genetic improvement of this long-lived, economically important organism.

## Introduction

Rust fungi cause some of the most important crop and tree diseases worldwide. In *Populus* species (poplar trees), leaf rust disease is caused by several species of *Melampsora*. Severe poplar leaf rust infections decrease photosynthetic capacity, reduce biomass, and increase susceptibility to additional pathogens [1]. Reductions in dry weight and fiber volume of *P. deltoides x P. balsamifera* ‘Northwest’, have been estimated at 57% and 65%, respectively [2]. In North America, hybridization of rust species, *M. occidentalis* and *M. medusae*, has produced a new rust pathogen, *M.* ×*columbiana*. This hybrid rust has demonstrated high pathogenic diversity. In an initial collection 13 pathotypes (race with unique virulence to specific hosts) were identified. Furthermore, host resistance loci to pathotypes *Mxc3* and *Mxc4* map to unique chromosomes in poplar and are race-specific [3], [4].

Rust virulence and poplar resistance interact in a classical gene-for-gene model, where host R gene recognition of a pathogen avirulence gene is necessary for resistance [5].The pathogenic diversity of *Melampsora* is facilitated by obligate sexual reproduction on alternative hosts (*Larix* species) during winter and migration of wind-dispersed spores. Previous studies of *M. medusae* populations suggest that inoculum source and genetic composition vary from year to year outside the range of alternative host sympatry [6]. This is also evident in *M. ×columbiana* populations in the coastal Pacific Northwest, where *Larix* spp. are not native and rust pathotypes varied across years at single locations [4].

Poplar leaf rust resistance has been extensively studied using the European counterpart, *M. larici-populina* [7]–[11]. Bi-parental linkage mapping has identified major resistance loci that map to nucleotide binding site – leucine rich repeat (NBS-LRR) R genes on chromosome 19 [7]–[9], [11]. During the mid-1990s, *P. trichocarpa* × *P. deltoides* ‘Beaupré’ was bred for rust resistance and then exclusively planted in European plantations. Yet, a rust epidemic occurred after the R gene was defeated by the evolution of virulence factor 7 [1], [12]. In wheat, NBS-LRRs have also conferred race-specific resistance to stem rust; however, association mapping has recently been applied to validate non-R gene non-race-specific resistance loci [13].

Association mapping in plants has the capability to precisely identify a greater number of functional variants which explain smaller proportions of the phenotypic variance than traditional linkage analysis [14]. The development of genomic resources necessary for association analysis in *Populus* species have been facilitated by its value to wood and paper industries and its potential as a biofuels feedstock. With large unstructured populations and wide phenotypic diversity, wood traits in *P. trichocarpa* have begun to be studied via association analysis [15]. Here, we report the first multi-year association analysis of poplar leaf rust resistance SNPs in *P. trichocarpa*.

## Methods

### Plant Material and Phenotyping

The ramets of 456 genotypes of native black cottonwood were collected from the common garden of British Columbia Ministry of Forest, Lands and Natural Resource Operations (MOFLNRO) at Surrey, BC in March of 2008. MOFLNRO collected these native poplar genotypes from 136 provenances from 44.00 degrees north latitude (Oregon USA) to 59.34 degrees north latitude of (Alaska USA) under the authority of Dr. Alvin Yanchuk, Technical Advisor for the Tree Improvement Branch (Victoria, BC Canada). In June of 2008, four replicates of each genotype were planted in a common garden situated at the University of British Columbia in Vancouver, Canada (49.27 degree north latitude). Replicates were planted in a completely randomized design with 1.5×1.5 meter spacing. In 2008 and 2009, fields were watered daily by rainfall or drip irrigation. No fertility or soil amendments were applied at any time. In 2009, 2010, and 2011 natural infection from *Melampsora* ×*columbiana* was scored visually on the basis of pustules present on the leaves. Ratings were taken on a 0–4 scale where (0) = no pustules, (1) = less than five pustules per leaf on less than five leaves, (2) = less than five pustules per leaf on more than five leaves, (3) = more than five pustules per leaf on more than five leaves, (4) = more than five pustules on all leaves. Ratings were taken once a week for 11 consecutive weeks (Julian Day 200–279). Ratings were used to calculated area under the disease curve (AUDPC) using the following equation [16]:



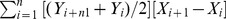
(1)where, *Y* *_i_* is the disease rating at the *i* ^th^ observation, *X_i_* is the time at the *i* ^th^ observation, and *n* is the total number of observations. Genotypes with missing scores from all four replicates were removed from the study and reduced the population to 412 genotypes. Date of bud set was taken concurrently with disease ratings and varied widely (data not shown). Host age can interact with disease resistance [17] thus, AUDPC scores were adjusted for bud set using ANCOVA in Minitab v16 (Minitab® Statistical Software). Finally, all adjusted AUDPC scores were transformed for normalization using the following equation:



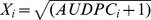
(2)


Data normality was tested using Lilliefors (Kolomorov-Smirnov) test in R package “nortest”.

### SNP Genotyping

We genotyped a total of 456 clones of the *P. trichocarpa* population using an Illumina Infinium® genotyping array with a set of 34,131 SNPs in 3,543 candidate genes. The 34K SNP genotyping array we employed was designed to take linkage disequilibrium (LD) into account. SNPs in any given candidate gene represented on the array were chosen to “tag” as many other target SNPs as possible (based on LD calculations), with a SNP density of approximately 1–2 SNPs per candidate gene kb [18]. We eliminated SNPs with: i) minor allele frequency below 0.05, ii) more than 10% missing values, and iii) an Illumina GeneTrain score below 0.5. These three selection criteria reduced the number of SNPs to 29,355. These remaining SNPs were used in all subsequent analyses.

### Population Structure

To fit population structure effect, we used a subset of 899 randomly selected SNPs distributed across all 19 chromosomes with complete information (i.e., no missing data) and meeting HWE expectation (tested using “HWChisq” function implemented in “HardyWeinberg” R package [19]. Population fit was done by performing principal component analysis (PCA) in TASSEL [20] and 263 principal components accounting for 90% of the total variance in the SNP data were retained for further determination of their impact. Principal components affecting AUDPC in each year were selected through regression in a stepwise manner using the function “stepwise” implemented in R package “Rcmdr” with “backward” direction and Bayesian information criterion “BIC” as the selection criterion.

Kinship matrix was calculated using the above mentioned 899 SNPs in SPAGeDi [21]. All negative values were set as zero and diagonal elements were set to one [22]. **Q** matrix and F_ST_ were calculated using GENELAND software with a subset of 200 SNPs randomly selected from the 899 used in PCA. Pearson’s product moment correlations for latitude, AUDPC, ***Q*** matrix, and PC1 were calculated in R package “Rcmdr”.

### Association Analysis

We applied a two-step approach to analyze SNP-AUDPC association [23]. First, a simple linear regression with AUDPC in each year and every SNP was used to pre-select SNPs with the following equation:



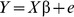
(3)where, *Y* is the observations vector, *β* is the fixed effects of population mean and SNP effect vector, *X* is the incidence matrix assigning fixed effects to observations, and *e* is the residual effect. SNP genotypes were coded as 0, 1, and 2 for common allele homozygote, heterozygote, and rare allele homozygote, respectively. SNPs with significant effect (*P*<0.001) were included in the second analysis.

Finally, the selected principal components were included in a regression model along the screened SNP individually as follows:



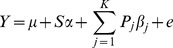
(4)where, *Y* is vector of measurements, is the population mean, is the SNP effect, the term represents the effect of selected principal components resulting from backward stepwise selection procedure (above), and *e* is the residual effect. Association analysis was performed in TASSEL [20] employing the GLM procedure. Permutated *P* value was calculated in TASSEL with 1,000 permutations. The correction for multiple testing was applied at α <0.05. Cumulative effect of SNPs within each year was calculated using method described by Ingvarsson *et al.* [24]. Pairwise LD plots was calculated using the “LDheatmap” function implemented in the LDheatmap R package [25].

### Variance Components and Spatial Analysis

A REML-mixed linear model was used to estimate variance components in SAS and test the effect of clone, year, and clone ×year interaction as follows:



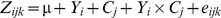
(5)where, *Z* is vector of measurements (AUDPC) in the *i*th year, of the *j*th clone, in the *k*th ramet, *µ* is the population mean, *Y* *_i_* = effect of the *i*th year, *C_j_* = effect of the *j*th clone, *Y* *_i_*×*C_j_* = effect of the *i*th year × the *j*th clone interaction, *e* is the residual effect. Broad-sense heritability in each year was calculated using methods described in Lynch & Walsh [26].

In each year the mixed linear model implemented in ASReml [27] was used to plot the residuals to their location in the field as follows:



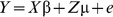
(6)where *Y* is vector of measurements, *β* and *µ* are vectors of fixed (intercept and population) and random (genotypic values) effects assuming *U∼N(0,* *)* and *Var(µ) = I*, *e* is vector of residual effects assuming *E∼N(0,* *)* and *Var(e) = I* where *I* is identity matrix containing 1′s on diagonal and 0′s at diagonal-off elements, and *X* and *Z* are index matrices assigning both fixed and random effects to measurements. ***Q*** matrix from GENELAND analysis was used to fit population structure effect.

## Results

### Disease Analysis

To identify SNPs that confer non-race-specific resistance to *M.* ×*columbiana*, we performed association analysis on 412 unrelated *P. trichocarpa* genotypes from a North American provenance trial ranging from Alaska to Oregon. Poplar leaf rust severity was scored (0–4 worst) on natural infection in a replicated (ramets = 4) common garden experiment where ratings were taken over 11 continuous weeks (Julian days 200 – 279) in each of three consecutive years. Rust severity ranged from zero rust pustules after 11 weeks (complete resistance) to 100% of the leaves covered after four weeks (Julian day 229).

Area under the disease progress curve (AUDPC) was calculated from the disease ratings over time. Previously, AUDPC had the highest broad-sense heritability (*H* ^2^ = 0.69) among four other measures of *Melampsora* resistance in growth chamber assays with artificial inoculations [28]. We estimated broad-sense heritability for AUDPC at *H* ^2^ = 0.72, 0.65, and 0.58 for each of the three years, respectively. Analysis of variance indicated that clone and clone × year interaction were significant, while year was not significant (Table 1). Spatial analysis of the experimental plot also demonstrated a change in the pattern of infection across years (Fig. S1).

**Table 1:** Analysis of variance (ANOVA) testing the effect of clone, year, and clone × year interaction of AUDPC.

Sources of Variance	Estimate	St. Error	Z value	Pr>Z
Clone	44.6077	3.0644	14.56	<0.0001
Year	27.9939	28.0179	1	0.1589
Clone x Year	5.1717	0.5507	9.39	<0.0001
Residual	27.2343	0.5616	48.5	<0.0001

### Population Structure and Association Analysis

**Figure 1:**
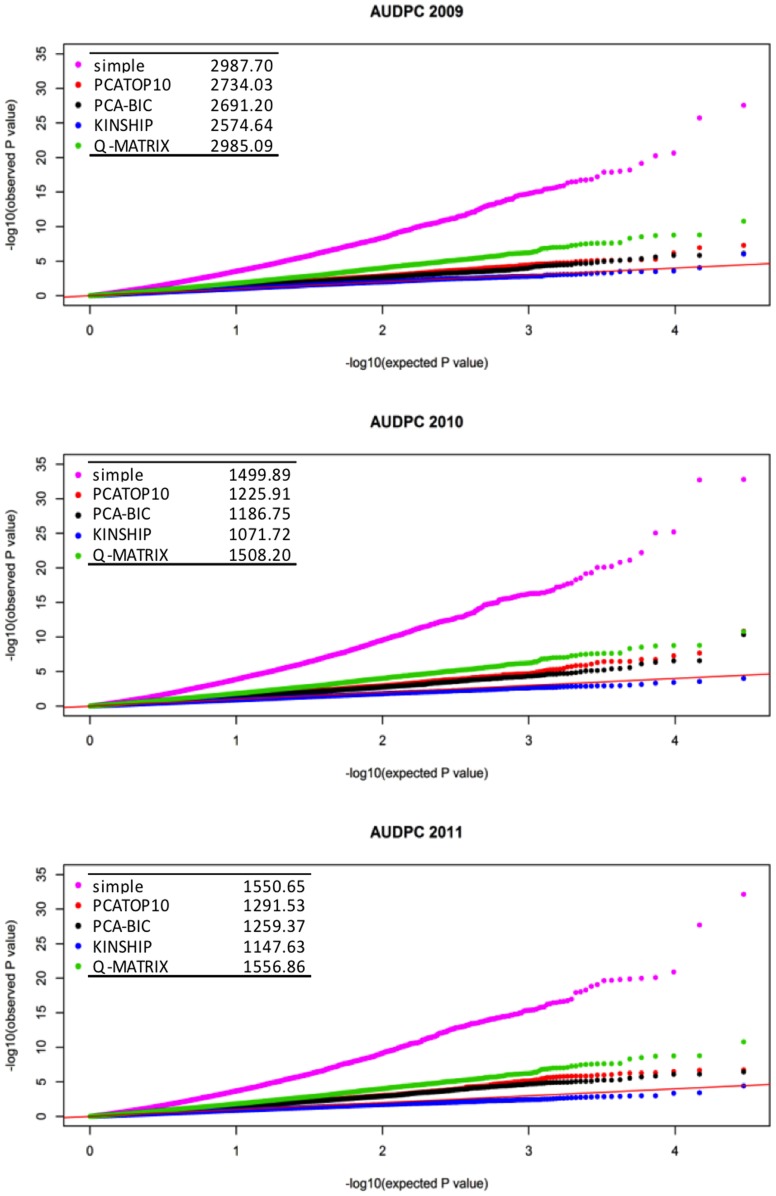
Quantile-quantile plots of expected and observed *P* values evaluating the type-1 error in a simple model (simple), the top ten principal components (PCA-TOP10), BIC selected PCs (PCA-BIC), the K model (KINSHIP), and the *Q* model (*Q* MATRIX) with goodness of fit test using Bayesian Information Criterion for 2009, 2010, and 2011 (top to bottom).

Analysis of population structure was tested using GENELAND software [29]. An uncorrelated allele frequency model did not detect any population structure while a correlated allele frequency model revealed three sub-populations with weak systemic structure (F_ST_ <0.0227) consistent with our previous results [18], [30]. Components of the population structure also displayed strong correlation to AUDPC and latitude (Table 2; Fig. S2). Due to this correlation of phenotype and population structure, the trait-SNP simple model (simple linear regression) produced 941, 1220, and 1093 significant associations at *P*<1.72×10^−6^ in each of the three years, respectively (data not shown) and a prodigious inflation of type-1 error (Fig. 1).

**Table 2:** Correlation coefficients of the population structure, latitude, and AUDPC in each year.

	Latitude	Q1	Q2	Q3	PC1	AUDPC09	AUDPC10
Q1	−0.37016						
	<0.0001^a^						
Q2	−0.08427	−0.74541					
	0.0876	<0.0001					
Q3	0.65247	−0.49093	−0.2148				
	<0.0001	<0.0001	<0.0001				
PC1	−0.62464	0.65285	−0.17031	−0.73393			
	<0.0001	<0.0001	0.0005	<0.0001			
AUDPC09	0.73339	−0.42216	0.00914	0.60658	−0.67059		
	<0.0001	<0.0001	0.8533	<0.0001	<0.0001		
AUDPC10	0.67009	−0.53882	0.05547	0.71694	−0.73558	0.77123	
	<0.0001	<0.0001	0.2613	<0.0001	<0.0001	<0.0001	
AUDPC11	0.73398	−0.50088	0.09273	0.61266	−0.69105	0.79764	0.8213
	<0.0001	<0.0001	0.06	<0.0001	<0.0001	<0.0001	<0.0001

a *P* value of Pearson’s correlation coefficient (n = 412).

Q1, Q2, Q3 represent the three sub-populations revealed by GENELAND analysis where pairwise F_ST_ were calculated. Q1×Q2 = 0.0118, Q1×Q3 = 0.0226, and Q2×Q3 = 0.02. PC1 represents the first principal component used to correct for population stratification.

During association analysis, we tested the effects of ***Q*** matrix, PCA-based model that uses the first 10 PC’s (PCA-TOP10) [31] PCA-based model with PCs that affect AUDPC selected through a backwards step-wise regression (PCA-BIC), and kinship matrix [32]. In a goodness of fit test, kinship matrix had the lowest Bayesian Information Criterion (BIC) value; however in the 2010 and 2011 quantile-quantile plots (q-q plots), kinship matrix eliminated all of the expected associations. The PCA-BIC displayed the next best model fit without overcorrecting for structure (Fig. 1).

Association analysis was performed with 29,355 SNPs covering 3,543 genes in a linear regression with AUDPC using the PCA-BIC model in each year. In 2009, 2010, and 2011 a total of three, four, and three SNPs were significantly associated at *P*<1.46×10^−6^. Bonferroni correction for multiple testing was applied at α = 0.05/29,355 where *P* = 1.70×10^−6^. Single SNPs located in *PSEUDO RESPONSE REGULATOR7* (*PRR7*) and *IQ-DOMAIN32* (*IQD32*) were repeated in three and two years respectively. In addition, two SNPs in high linkage disequilibrium (LD) (R^2^>0.8) located in the intergenic region between *NITRATE TRANSPORTER2.1* (*NRT2.1*) and *NITRATE TRANSPORTER2.4* (*NRT2.4*) were also significant (Table 3).

**Table 3:** SNPs associated with AUDPC in 2009, 2010, and 2011.

Year	Scaffold	SNP	Gene Model	SNP location	Arabidopsis Best hit	Annotated Gene	*P* value	Permutated *P* value	Marker R^2^
2009	10	19215715	POPTR_0010s22230	exon	At5g02810.1	PRR7 (PSEUDO-RESPONSE REGULATOR 7)	7.33414E-07	0.003	0.0289
	14	3245282	POPTR_0014s04070	intron	At1g19330.1	unknown protein	1.45407E-06	0.003	0.0271
	14	3245414	POPTR_0014s04070	intron	At1g19330.1	unknown protein	1.45407E-06	0.003	0.0271
	12	1814218	POPTR_0012s02170	intron	At3g49220.1	pectinesterase family protein	4.44885E-06	0.012	0.0250
	12	1814164	POPTR_0012s02170	intron	At3g49220.1	pectinesterase family protein	5.76602E-06	0.018	0.0244
	5	23949327	POPTR_0005s25750	intergenic	At1g19870.1	IQD32 (IQ-domain 32)	7.86988E-06	0.025	0.0238
	2	13904004	POPTR_0002s18010	intergenic	no arabidopsis blast hit	unknown protein	9.02706E-06	0.030	0.0236
	10	1844266	POPTR_0010s01650	intergenic	At4g15900.1	PRL1 (PLEIOTROPIC REGULATORY LOCUS 1)	1.06875E-05	0.033	0.0253
	1	31118784	POPTR_0001s32810	intron	At4g13980.1	AtHSFA5; DNA binding/transcription factor	1.40977E-05	0.037	0.0226
2010	10	19215715	POPTR_0010s22230	exon	At5g02810.1	PRR7 (PSEUDO-RESPONSE REGULATOR 7)	4.49274E-11	0.001	0.0476
	5	23949327	POPTR_0005s25750	intergenic	At1g19870.1	IQD32 (IQ-domain 32)	2.71307E-07	0.003	0.0302
	5	10782555	POPTR_0005s13780	intergenic	At2g23760.1	BLH4 (BEL1-LIKE HOMEODOMAIN 4)	4.67804E-07	0.004	0.0292
	5	23952538	POPTR_0005s25750	exon	At1g19870.1	IQD32 (IQ-domain 32)	7.99477E-07	0.004	0.0281
	6	1402770	POPTR_0006s02140	intergenic	At4g15090.1	FAR1 (FAR-RED IMPAIRED RESPONSE 1)	3.6489E-06	0.016	0.0253
	6	1397889	POPTR_0006s02140	3′-UTR	At4g15090.1	FAR1 (FAR-RED IMPAIRED RESPONSE 1)	4.07883E-06	0.017	0.0250
	1	34721616	POPTR_0001s36210	exon	At3g27330.1	zinc finger (C3HC4-type RING finger) family protein	6.1898E-06	0.024	0.0244
	6	1399289	POPTR_0006s02140	exon	At4g15090.1	FAR1 (FAR-RED IMPAIRED RESPONSE 1)	7.28474E-06	0.029	0.0238
	9	10970414	POPTR_0009s13880	intergenic	At4g02390.1	APP (ARABIDOPSIS POLY(ADP-RIBOSE) POLYMERASE)	7.59421E-06	0.029	0.0239
	6	1402469	POPTR_0006s02140	intergenic	At4g15090.1	FAR1 (FAR-RED IMPAIRED RESPONSE 1)	8.05648E-06	0.031	0.0242
	14	10716774	POPTR_0014s14650	intron	At5g48560.1	basic helix-loop-helix (bHLH) family protein	1.1107E-05	0.041	0.0230
2011	9	1676227	POPTR_0009s01420	intergenic	At1g08090.1	PtNRT2.1 (NITRATE TRANSPORTER 2.1)	3.52242E-07	0.003	0.0342
	5	23949327	POPTR_0005s25750	intergenic	At1g19870.1	IQD32 (IQ-domain 32)	7.88901E-07	0.003	0.0308
	10	19215715	POPTR_0010s22230	exon	At5g02810.1	PRR7 (PSEUDO-RESPONSE REGULATOR 7)	8.34729E-07	0.003	0.0313
	9	1678826	POPTR_0009s01420	intergenic	At1g08090.1	PtNRT2.1 (NITRATE TRANSPORTER 2.1)	1.9236E-06	0.007	0.0308
	9	1606213	POPTR_0009s01330	exon	At3g45040.1	phosphatidate cytidylyltransferase family protein	2.96357E-06	0.008	0.0279
	9	1857142	POPTR_0009s01490	intergenic	At5g60720.1	unknown protein	4.83027E-06	0.014	0.0270
	8	4165833	POPTR_0008s06920	intergenic	At5g05610.1	AL1 (ALFIN-LIKE 1)	5.71705E-06	0.017	0.0266
	9	1676590	POPTR_0009s01420	3′-UTR	At1g08090.1	PtNRT2.1 (NITRATE TRANSPORTER 2.1)	5.74407E-06	0.017	0.0266
	143	2955	POPTR_0143s00200	exon	At5g60770.1	PtNRT2.4 (NITRATE TRANSPORTER 2.1)	5.74407E-06	0.017	0.0266
	2	13131622	POPTR_0002s17360	intergenic	no arabidopsis blast hit	unknown protein	7.73544E-06	0.022	0.0259
	2	4627286	POPTR_0002s06880	intron	At1g76900.1	AtTLP1 (TUBBY LIKE PROTEIN 1)	8.13072E-06	0.025	0.0259
	10	21451968	POPTR_0010s26100	5′-UTR	At3g54540.1	AtGCN4; transporter	8.23563E-06	0.025	0.0261
	17	12392905	POPTR_0017s12210	3′-UTR	At5g61430.1	ANAC100 (ARABIDOPSIS NAC DOMAIN CONTAINING PROTEIN 100)	1.02655E-05	0.035	0.0254
	9	1679212	POPTR_0009s01420	intergenic	At1g08090.1	PtNRT2.1 (NITRATE TRANSPORTER 2.1)	1.09575E-05	0.039	0.0252
	12	1811250	POPTR_0012s02170	intergenic	At3g49220.1	pectinesterase family protein	1.10236E-05	0.039	0.0253
	9	1679805	POPTR_0009s01420	intergenic	At1g08090.1	PtNRT2.1 (NITRATE TRANSPORTER 2.1)	1.16257E-05	0.040	0.0250
	8	8157244	POPTR_0008s12610	exon	At5g17350.1	unknown protein	1.1642E-05	0.040	0.0251
	6	1405713	POPTR_0006s02150	exon	At3g22170.1	FHY3 (FAR-RED ELONGATED HYPOCOTYLS 3)	1.22801E-05	0.041	0.0250
	9	2563210	POPTR_0009s01990	intron	At5g60690.1	REV (REVOLUTA)	1.28046E-05	0.043	0.0249
	8	8261867	POPTR_0008s12780	exon	At1g71010.1	phosphatidylinositol-4-phosphate 5-kinase family protein	1.31391E-05	0.046	0.0248

Permutated *P* value of α = 0.05 was used as the threshold for multiple testing corrections after SNP pre-selection.

Bonferroni correction threshold was applied at *P* = 1.70×10^−6^ without SNP pre-selection.

Arabidopsis best hit and annotated function is derived from BLAST results of poplar gene models in POPGENIE. R^2^ value explains the effect of each SNP on the phenotype.

**Figure 2:**
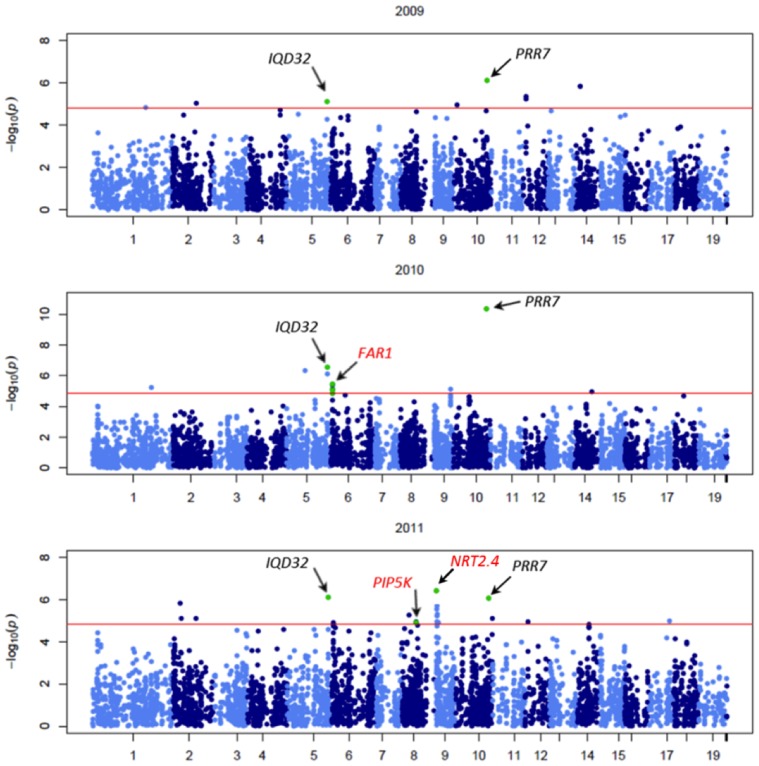
Manhattan plot of the results from association analysis for AUDPC in 2009, 2010, and 2011 (from top to bottom). The red line represents the *P* value (*P*<1.45×10^−5^, *P*<1.15×10^−5^, and *P*<1.35×10^−5^ in 2009, 2010, and 2011, respectively) corresponding to permutated *P* of α = 0.05 as the threshold for multiple testing corrections. SNPs repeated in time are highlighted in green and identified by gene name in black. SNPs within genes showing expression profile changes in response to *M. larici-populina* are highlighted in green and identified by gene name in red.

In the final analysis, we used a simple linear regression with AUDPC in each year to preselect SNPs and reduce the constraint of multiple testing correction. Significant SNPs at *P*<0.001 were selected and re-run in a linear regression with the PCA-BIC model to correct for population structure. In 2009, 2010, and 2011 a total of 9, 11, and 20 SNPs achieved experiment-wide significance at *P*<1.45×10^−5^, *P*<1.15×10^−5^, and *P*<1.35×10^−5^, respectively (Fig. 2; Table 3). Permutated p-value was used as correction for multiple testing at a threshold α <0.05. Individually, these SNPs explain 2.2–4.7% of the phenotypic variance. The cumulative effects of independent SNP associations explain 12.1, 14.2, and 19.6% of the phenotypic variance within each year, respectively (Table 3).

Single SNPs located in *PRR7* and *IQD32* were repeated in all three years (Table 1). In addition, SNPs in *FAR-RED IMPAIRED RESPONSE1* (*FAR1*), a phosphatidylinositol 4-phosphate 5-kinase (POPTR_0008s12780; *PIPK5*), and *NITRATE TRANSPORTER2.4* (*NRT2.4*) were associated in a single year. In host infection transcriptome analysis, these genes were differentially expressed in incompatible interactions with additional *Melampsora* species (unpublished data). Arabidopsis orthologs corresponding to genes housing these five SNPs indicate functions in host defense through transcriptome reprogramming, calcium and salicylic acid signaling, and tolerance to reactive oxygen species.

## Discussion

In prior descriptions of this population, growth traits and population stratification differentiated in a north to south pattern [15], [18], [30], [33], [34]. This differentiation may be driven by intense selection pressure for adaptation to day-length and physical barriers impeding gene flow [33]. Rust severity was also correlated to latitude. Rust aggressiveness can be reduced in below optimal temperatures (20°C) in both poplar leaf rust and wheat rust interactions [35], [36]. Cooler temperatures at northern latitudes may produce a weaker selection pressure for resistance than in the south, thus creating co-linearity of causal variants with population structure. Co-linearity of allele frequencies via correlation of phenotype and population structure inflates type-1 error in association studies; therefore, it is necessary to correct for the confounding effects of population structure.

Several approaches have been proposed to correct for structure. Yu *et al*. [22] proposed a unified mixed model which fits both population (***Q*** matrix) and familiar structure (kinship matrix) to precisely eliminate confounding factors. Consideration of a residual polygenic term fitting LD pattern across chromosomes in the model, which is usually fit solely by kinship matrix, has also improved the estimate of the genetic relatedness [37]–[39] especially in complex polygenic traits. Moreover, the kinship matrix itself is considered to fit both confounding factors efficiently [31], [40], [41]. In our study, GENELAND analysis indicated very weak stratification among three sub-populations (F_ST_ <0.0227). The use of ***Q*** matrix in the association model resulted in decreased fit and inflated type-1 error. Kinship matrix had the lowest BIC value in a goodness of fit test, suggesting that it is the best model to correct for the confounding structure; however the q-q plots indicated that kinship matrix eliminated the expected associations and overcorrected the model (Fig. 1).

Alternatively, Price *et al*. [42], [43] employed principal component analysis to improve the correction for population stratification and the confounding effects of phenotype – population structure correlation. They proposed using a fixed number of principal components (first 10) or ones selected on the basis of Tracy-Widom statistics [44] when admixture occurred in population regardless of their relationship to phenotype. Methods using a stepwise regression to select a set of SNPs [37], [45] or principal components [23], [46] have been suggested to fit the confounding structure and used as regressors in the final association analysis model. Novembre & Stephens [47] also indicated that inclusion of principal components not correlated with the trait may reduce power. In our study, the inflation of significant associations and the goodness of fit in the PCA-TOP10 model in comparison to the PCA-BIC model further supports this hypothesis (Fig. 1).

We also used SNP pre-selection to reduce the constraint of multiple testing corrections on inflation of false negative associations [23], [48]. We reason that elevating the pre-selection threshold from *P<*0.05 to *P<*0.001 would remove erroneous SNPs that would have been selected via the correlation of AUDPC and population stratification and thus increase type-2 error. In 2009, pre-selection at *P<*0.05 would have selected 10,828 SNPs for AUDPC, where *P<*0.001 reduced the SNP selection to 3,905 (data not shown). Moreover, SNP associations in *FAR1* and *PIPK5* were only achieved via SNP pre-selection but correlated to rust resistance through transcriptome analysis during incompatible poplar leaf rust interactions.

Associated SNPs within *PRR7*, *IQD32*, and *PIPK5* were in low LD with the adjacent SNPs. Thus, these SNPs may be causative variants or in high LD with the unrepresented causative SNP. Conversely, several SNPs within the neighboring gene pairs; *FAR1* and *FHY3*, and *NRT2.4* and *NRT2.1*, respectively, were in high LD which convolutes the elucidation of the true causative SNP.

Scaffold_10_ 19,215,715 is a non-synonymous polymorphism in the fifth exon of a sequence orthologous to *PRR7* (Fig. S3). In Arabidopsis, *PRR7* is a gene within a small family of circadian clock gene transcription factors [49]; however, it was not associated with phenological traits in this population (personal communications, Athena McKown). The *prr7* loss of function mutants has an ambiguous phenotype, but double and triple mutants accentuate the *prr5* and *prr9* single mutant phenotypes: arrhythmia with increased hypocotyl elongation, leaf number, and days to flowering [49]. More recently, *PRR7* was down-regulated in response to chitooctaose (chitin oligomer; chitin is a component of fungal cell walls). These results would suggest a role of *PRR7* transcriptional regulation during host infection [50].

Scaffold_5_23949327 is located 1,826 bp downstream from the 3′UTR of *IQD32* (Fig. S4). This gene is orthologous to a calmodulin binding protein that serves as an integral component of Ca^2+^/calmodulin signaling. In Arabidopsis and rice, *IQD* gene family members share as many as three calmodulin binding motifs IQ, 1-5-10, and 1-8-14. While *IQD* gene function has not been well characterized, *IQD1* has been shown to function in defense response to herbivory [51].

These single SNPs within *PRR7* and *IQD32* were associated in all three years. Bi-parental linkage mapping has identified major resistance loci that confer race-specific resistance to *M.* ×*columbiana* [4]. In our study, ANOVA and spatial analysis suggest the pathogenicity of the rust population varied across the three years. This is consistent with the reproductive biology of the rust where the non-overlap of poplar-alternative host ranges would affect the genetic composition of the rust population in time. Therefore, we propose that SNP-associations replicated in time and in the diversity of *M. ×columbiana* across the three years confer non-race-specific resistance.

**Figure 3:**
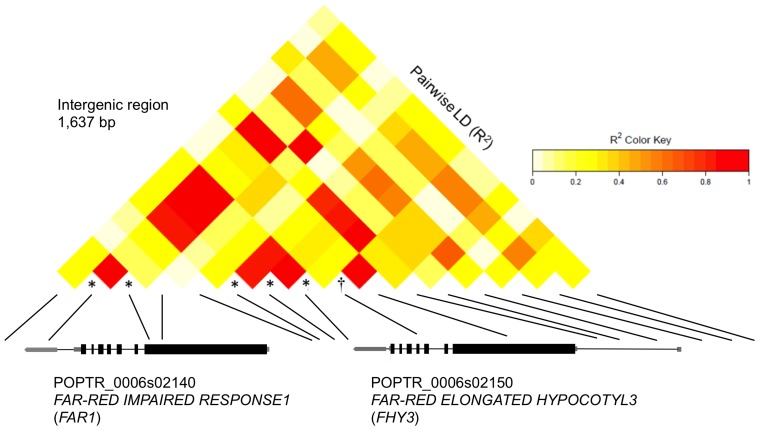
Pairwise linkage disequilibrium plot of *FAR-RED IMPAIRED RESPONSE1* and *FAR-RED ELONGATED HYPOCOTYL3* with gene structures. SNPs significant in 2010 are indicated with an asterisk; SNPs significant in 2011 are indicated with a cross. Scaffold_6_ 1402770 in the intergenic region had the highest significance (*P = *3.64×10^−6^) and explained 2.5% (R^2^ = 0.025) of the phenotypic variance in 2010.

Numerous signals within *FAR1* were also significant in 2010, but not repeated in time (Fig. 3). A homolog of *FAR1*, *FAR-RED ELONGATED HYPOCOTYL3* (*FHY3*), is a clock gene that indirectly mediates the phytochrome A response, but has additional functions. In Arabidopsis, *fhy3* mutants regulate plant architecture and abiotic stress tolerance through suppression of axillary bud outgrowth and repressed leaf growth with decreased tolerance to oxidative stress. Loss of function mutants in *far1* and *revoluta* (*REV*), a leucine-zipper transcription factor, enhance the *fhy3* phenotype [52]. In 2011, scaffold_6_1405713 within *FHY3* and scaffold_9_2,563,210 within *REV* were also significant (Table 3). These signals were not repeated in time; however, *FAR1* expression was increased 2-fold 96 hours after inoculation with *M. larici-populina* (unpublished data).

Likewise, scaffold_8_8261867 in the 12 exon of POPTR_0008s12780; encoding a phosphatidylinositol 4-phosphate 5-kinase (*PIP5K*), was significant in 2011 (Fig. S5) and has been previously implicated in resistance to *Melampsora*. In Arabidopsis, lower expression of *PIP5K*s leads to accumulation of the raffinose family oligosaccharides that act as osmoprotectants and antioxidants and protect mitochondria and chloroplasts from stress-induced production of reactive oxygen species [53]. In resistant *P. trichocarpa* × *deltoides*, this *PIP5K* gene was shown to have more than a 2-fold decrease in its expression 48 hours after inoculation with *M. larici-populina* [54].

**Figure 4:**
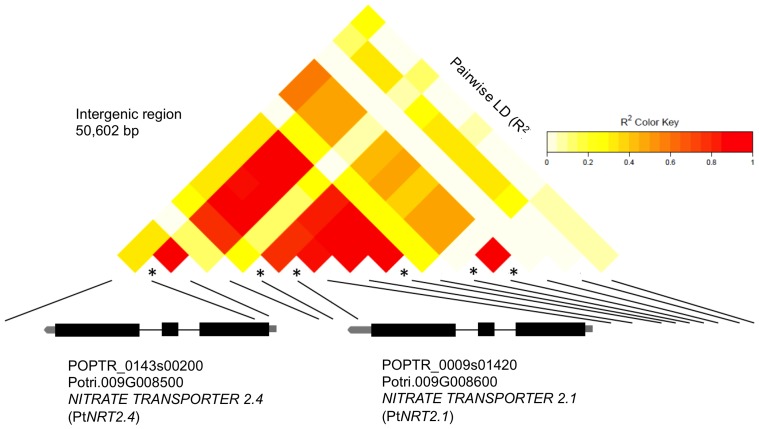
Pairwise linkage disequilibrium plot of *NITRATE TRANSPORTER2.1* and *NITRATE TRANSPORTER2.4* with gene structures. SNPs significant are indicated with an asterisk. Scaffold_9_ 1676227 in the intergenic region had the highest significance (*P = *3.52×10^−7^) and largest effect on AUDPC in 2010 (R^2^ = 0.034). Scaffold_143_2955 was annotated to Potri.009G008500 in JGI *Populus trichocarpa* genome v3.

The SNP at scaffold_143_2955 encodes a non-synonymous mutation in the third exon of *NRT2.4* and is in high LD (R^2^>0.8) with three other associated SNPs in the neighboring *NRT2.1* (Fig. 4). These genes are orthologous to the nitrate transporter At*NRT2.1*. Nitrate transporters are transmembrane proteins that primarily function in nitrate transport; however, they also function as environmental signal receptors and regulators of biotic and abiotic stress pathways. Recently, it was reported that the mutant *nrt2* that lacks the function of both AtNRT2.1 and the adjacent AtNRT2.2 shows decreased susceptibility to *Pseudomonas syringae*. The decrease in susceptibility is coordinated through an earlier and more robust induction of salicylic acid and up-regulation of defense genes *PR1* and *PR5* [55]. In poplar, *NTR2.4* was down-regulated in incompatible interactions of *P. deltoides* with *M. occidentalis* and *M. larici-populina* (unpublished data). The change in expression levels of genes housing associated SNPs during incompatible poplar leaf rust interactions further implicates a functional role in host defense.

In summary, we identified two independent loci that were strongly associated with host defense to *M. ×columbiana* and through repetition in time confer non-race-specific resistance. Furthermore, three other associated loci have been correlated to poplar leaf rust resistance through transcriptome analysis and may form a functional network with additional genes involved in tolerance to reactive oxygen species. In this long-lived ecologically and economically important tree species, these associations lay the foundation to more efficient breeding of durable disease resistance.

## Supporting Information

Figure S1 **Spatial distribution of residuals for AUDPC in each year. The scale of residuals ranges from −20 (low disease) to 20 (high disease).**
(TIFF)

Figure S2 **Population structure estimates and geographical distribution of each sampled tree (n = 412).** Colors designate the three sub-populations detected using GENELAND analysis (***Q*** matrix).
(TIFF)

Figure S3 **Pairwise linkage disequilibrium plot of** ***PSEUDO-RESPONSE REGULATOR7*** **and gene structure.** Scaffold_10_19215715 is indicated with an asterisk. In 2009, 2010, and 2011 (from left to right) each box plot shows the lower quartile, the median, and the upper quartile values, and the whiskers show the range of the phenotypic variation in the population.
(TIF)

Figure S4 **Pairwise linkage disequilibrium plot of** ***IQ-DOMAIN32*** **and gene structure.** Scaffold_5_23949327 is indicated with an asterisk. In 2009, 2010, and 2011 (from left to right) each box plot shows the lower quartile, the median, and the upper quartile values, and the whiskers show the range of the phenotypic variation in the population.
(TIF)

Figure S5 **Pairwise linkage disequilibrium plot of** ***PHOSPHATIDYLINOSITOL-4-PHOSPHATE 5-KINASE*** **and gene structure.** Scaffold_8_ 8261867 is indicated with an asterisk. In 2011, the box plot shows the lower quartile, the median, and the upper quartile values, and the whiskers show the range of the phenotypic variation in the population.
(TIF)
