# Supplementary figures and images for: Association Analysis Identifies Melampsora ×columbiana Poplar Leaf Rust Resistance SNPs
Source: PLoS One. 2013 Nov 13;8(11):e78423. doi: 10.1371/journal.pone.0078423 (PMC3827267; doi:10.1371/journal.pone.0078423)

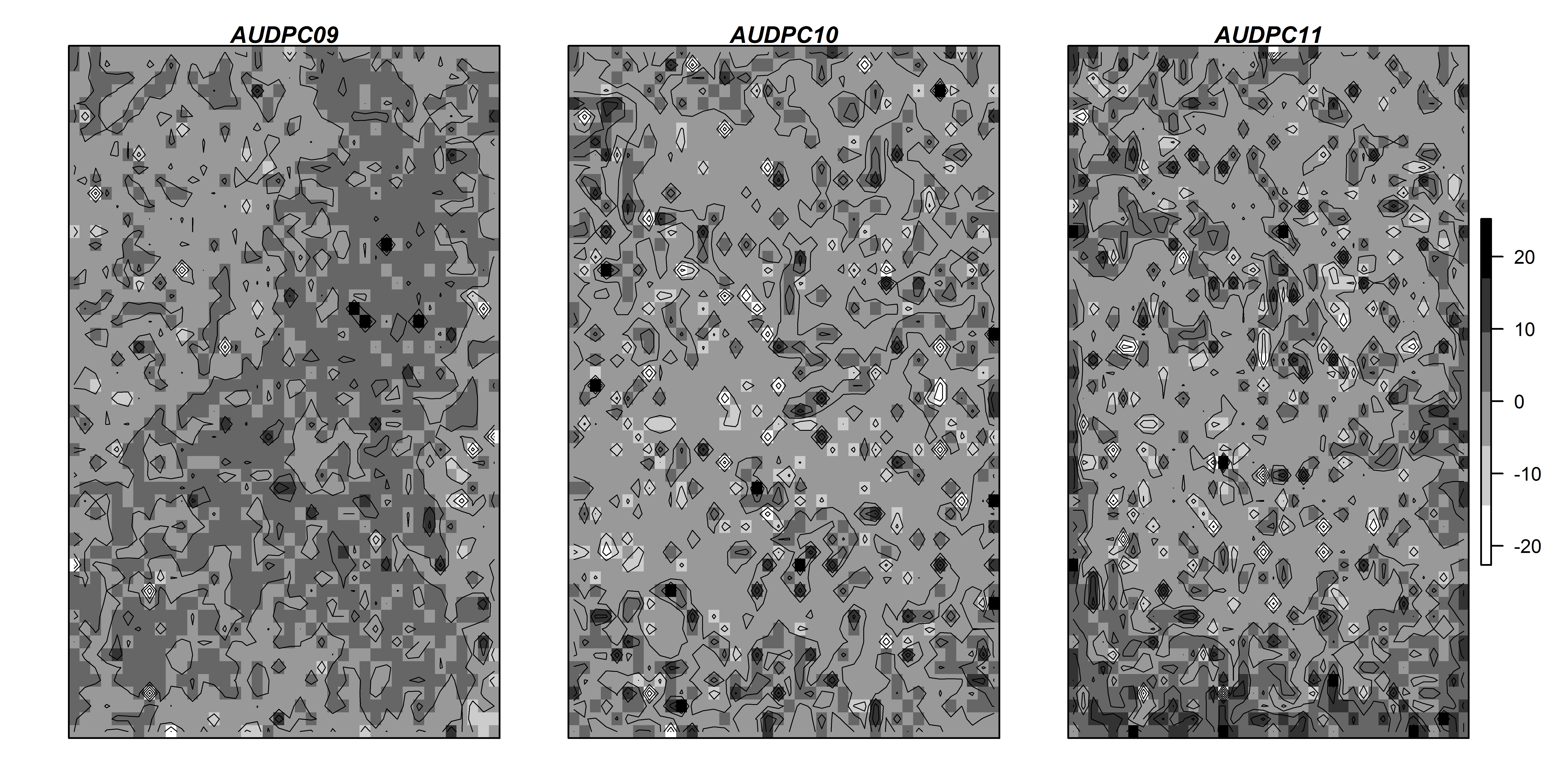

Supplement: Figure S1 — Spatial distribution of residuals for AUDPC in each year. The scale of residuals ranges from −20 (low disease) to 20 (high disease). (TIFF) [file pone.0078423.s001.tif]

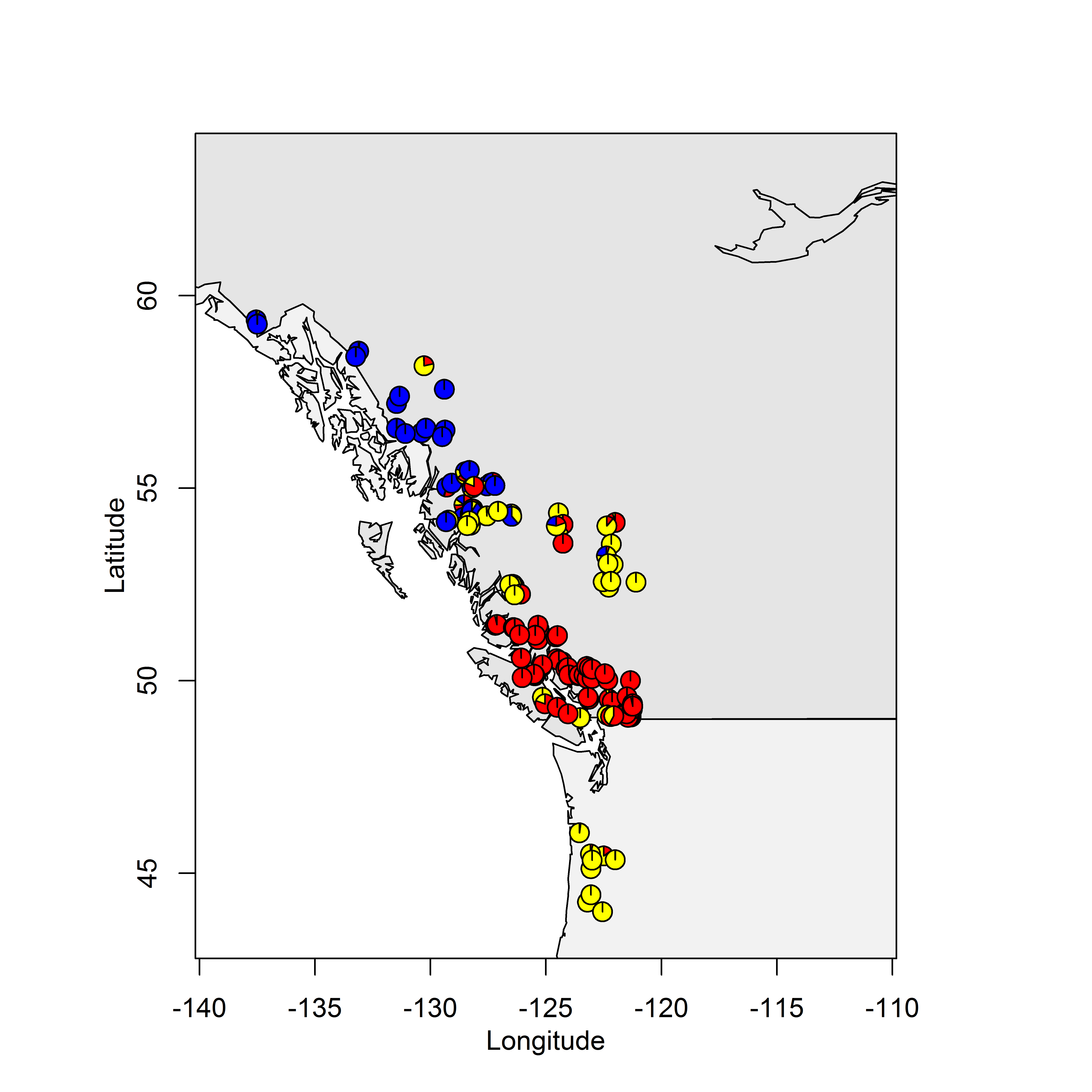

Supplement: Figure S2 — Population structure estimates and geographical distribution of each sampled tree (n = 412). Colors designate the three sub-populations detected using GENELAND analysis (Q matrix). (TIFF) [file pone.0078423.s002.tif]

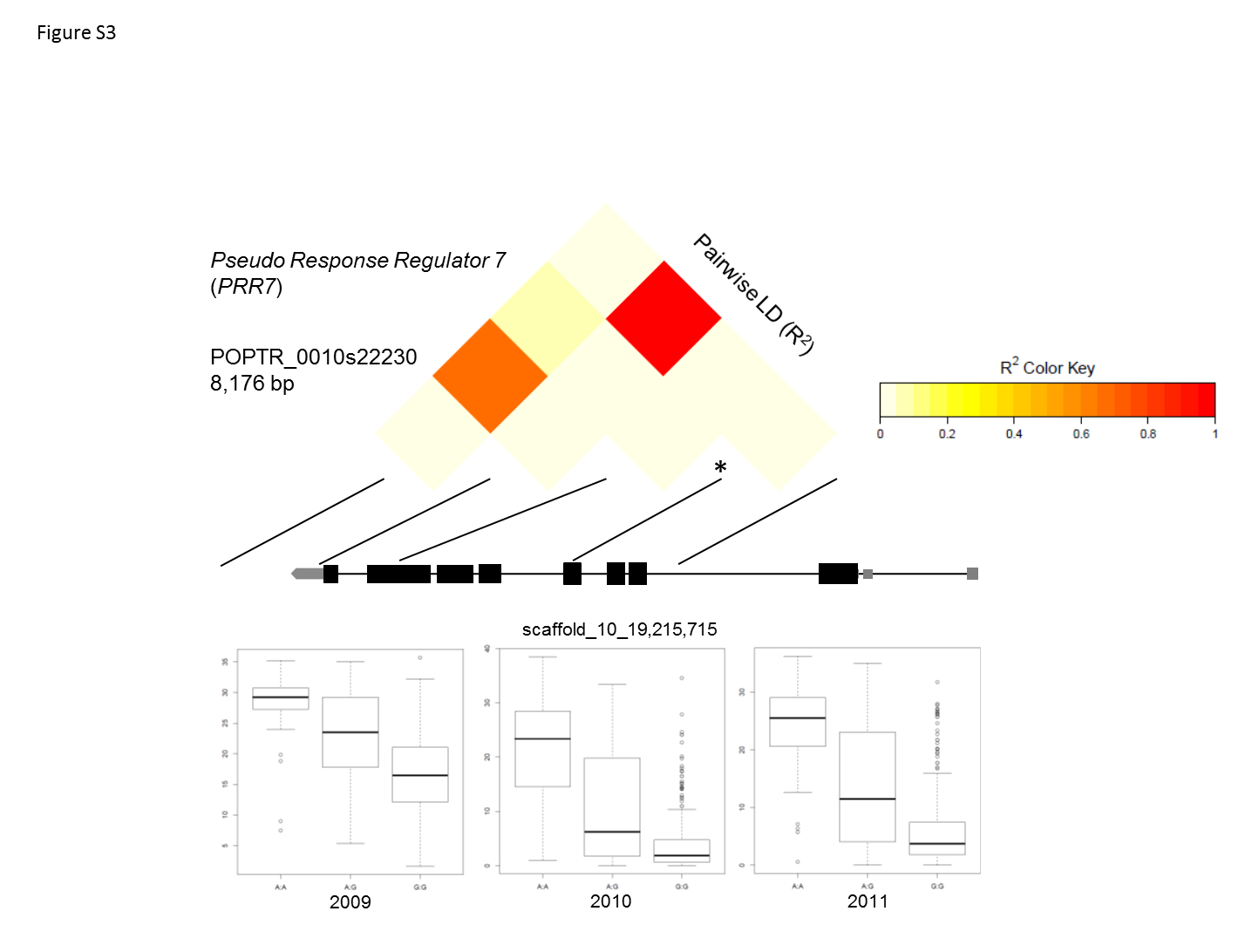

Supplement: Figure S3 — Pairwise linkage disequilibrium plot of PSEUDO-RESPONSE REGULATOR7 and gene structure. Scaffold_10_19215715 is indicated with an asterisk. In 2009, 2010, and 2011 (from left to right) each box plot shows the lower quartile, the median, and the upper quartile values, and the whiskers show the range of the phenotypic variation in the population. (TIF) [file pone.0078423.s003.tif]

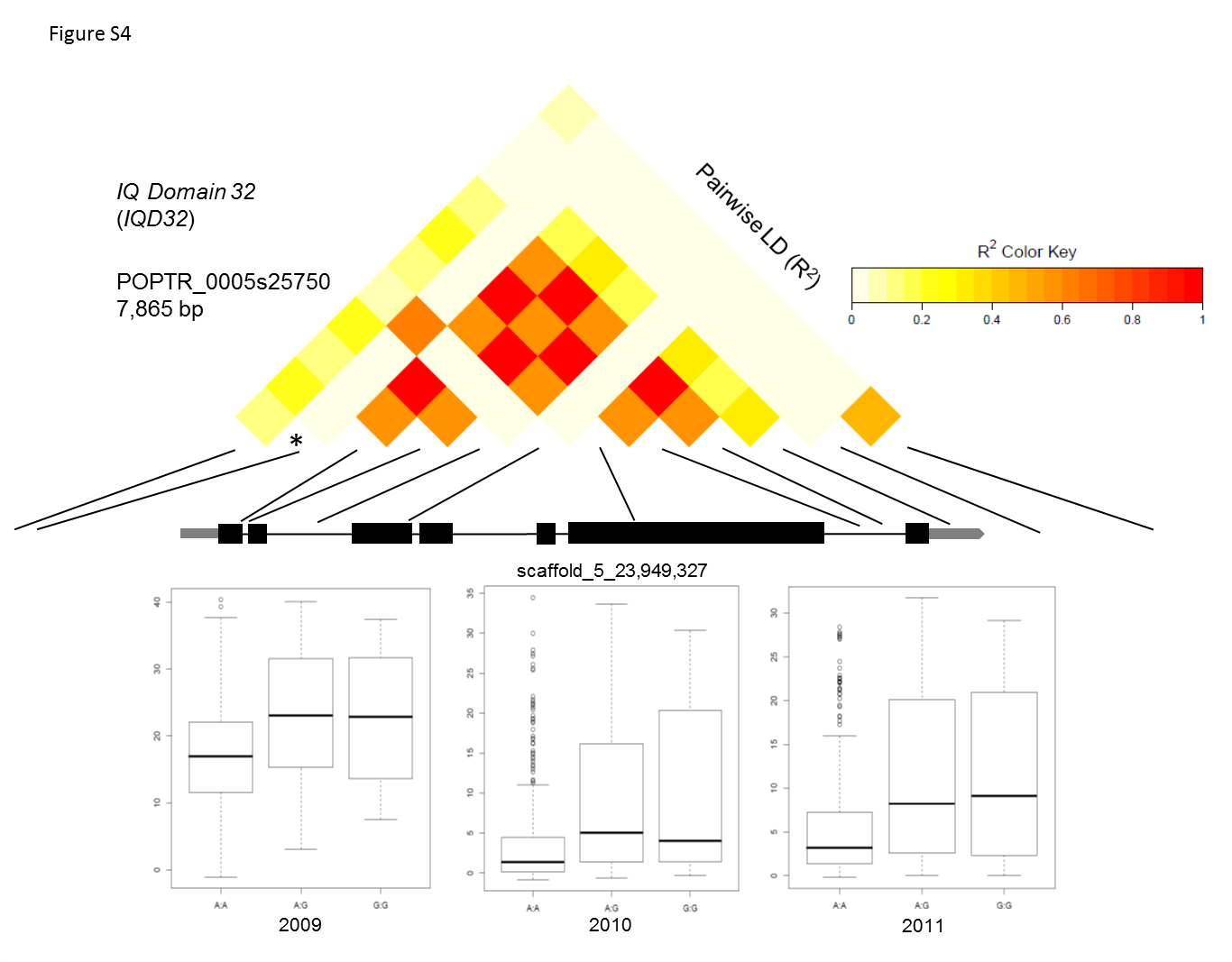

Supplement: Figure S4 — Pairwise linkage disequilibrium plot of IQ-DOMAIN32 and gene structure. Scaffold_5_23949327 is indicated with an asterisk. In 2009, 2010, and 2011 (from left to right) each box plot shows the lower quartile, the median, and the upper quartile values, and the whiskers show the range of the phenotypic variation in the population. (TIF) [file pone.0078423.s004.tif]

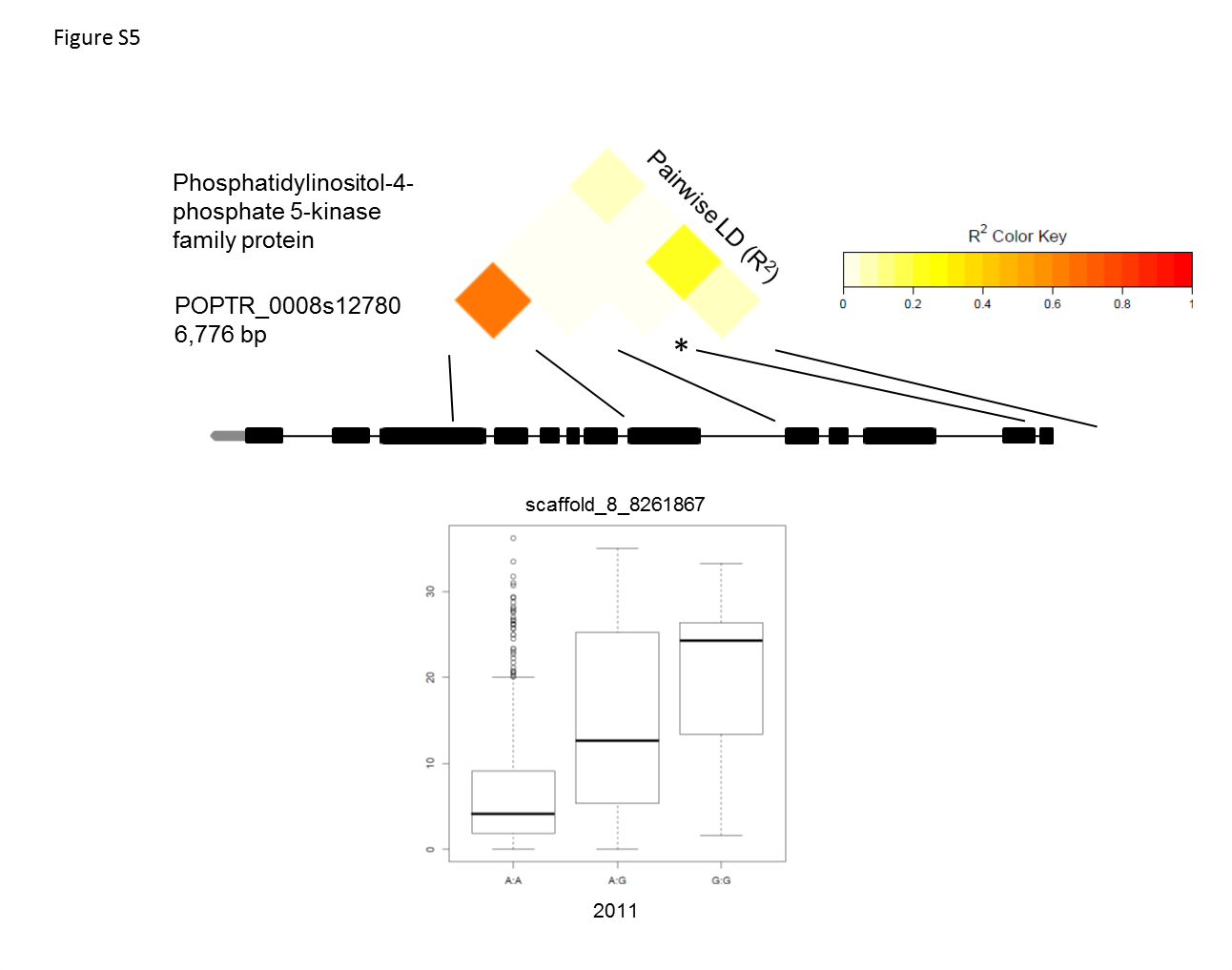

Supplement: Figure S5 — Pairwise linkage disequilibrium plot of PHOSPHATIDYLINOSITOL-4-PHOSPHATE 5-KINASE and gene structure. Scaffold_8_ 8261867 is indicated with an asterisk. In 2011, the box plot shows the lower quartile, the median, and the upper quartile values, and the whiskers show the range of the phenotypic variation in the population. (TIF) [file pone.0078423.s005.tif]
